# Mutation and immune profiling of metaplastic breast cancer: Correlation with survival

**DOI:** 10.1371/journal.pone.0224726

**Published:** 2019-11-06

**Authors:** Michelle Afkhami, Daniel Schmolze, Susan E. Yost, Paul H. Frankel, Andrew Dagis, Idoroenyi U. Amanam, Milhan Telatar, Kim Nguyen, Kim Wai Yu, Thehang Luu, Raju Pillai, Patricia A. Aoun, Joanne Mortimer, Yuan Yuan

**Affiliations:** 1 Department of Pathology, City of Hope Comprehensive Cancer Center, Duarte, CA, United States of America; 2 Department of Medical Oncology & Therapeutic Research, City of Hope Comprehensive Cancer Center, Duarte, CA, United States of America; 3 Department of Biostatistics, City of Hope Comprehensive Cancer Center, Duarte, CA, United States of America; 4 Department of Clinical Pharmacy, City of Hope Comprehensive Cancer Center, Duarte, CA, United States of America; Avera Research Institute, UNITED STATES

## Abstract

The goal of this study is to characterize the genomic and immune profiles of metaplastic breast cancer (MpBC) and identify the association with survival through an analysis of archived tumor tissue. A next-generation sequencing-based mutational assay (Onco-48) was performed for 21 MpBC patients. Clinicopathologic characteristics were captured, including relapse free survival (RFS) and overall survival (OS). Immunohistochemistry (IHC) for CD3, CD4, CD8, and programmed death-ligand 1 (PD-L1) was also performed. Recurrence free survival (RFS) at 5 years was 57% (95% CI 0.34–0.75) and overall survival (OS) at 5 years was 66% (95% CI 0.41–0.82). The most commonly altered genes were *TP53* (68.4%, 13/19), *PIK3CA* (42.1%, 8/19), and *PTEN* (15.8%, 3/19. For patients with *PIK3CA* mutations, RFS and OS were significantly worse than for those without (HR 5.6, 95% CI 1.33–23.1 and HR 8.0, 95% CI 1.53–41.7, respectively). Cox regression estimated that PD-L1 expression was associated with worse RFS and OS (HR 1.08, 95% CI 1.01–1.16 and HR 1.05, 95% CI 1.00–1.11, respectively, for an absolute increase in PD-L1 expression of 1%). In conclusion, *PIK3CA* mutation and *PD-L1* expression confer poor prognosis in this cohort of patients with MpBC.

## Introduction

Metaplastic breast cancer (MpBC) is a rare malignancy which accounts for 0.05–5% of all breast cancers [1, 2]. MpBC is defined by differentiation of the neoplastic epithelium to a non-glandular component, typically either squamous or mesenchymal (e.g. spindle cell, osseous, or chondroid). These cancers are subdivided into groups according to the 2012 WHO Classification of Tumors of the Breast: squamous cell carcinoma, spindle cell carcinoma, mixed squamous and spindle cell carcinoma, spindle cell and mesenchymal, or mesenchymal [3]. The conventional biomarkers of estrogen receptor (ER), progesterone receptor (PR), and human epidermal growth factor receptor 2 (HER2) are usually not expressed in metaplastic breast cancer (i.e., they are “triple negative” breast cancers). Initial gene expression profiling studies demonstrated that MpBC is of basal-like breast cancer [4]. Further analysis has classified MpBC into the “claudin-low” subtype based on mRNA expression profiling [5].

Clinically, MpBC is an aggressive form of breast cancer. Patients present with a more advanced stage and have a greater risk of local recurrence and a worse prognosis compared with conventional invasive ductal carcinoma [6]. The disease is often resistant to chemotherapy, possibly due to complex tumor genetics that results in phenotypically diverse histology and intratumoral heterogeneity [6]. Because of the rarity and heterogeneous nature of metaplastic cancers, there are no randomized controlled trials to inform treatment decisions. Treatment is generally determined by the dominant cell population. Next-generation sequencing (NGS) provides a unique opportunity to understand the underlying biology of cancer. NGS can also assist clinicians in identifying potential biomarkers for risk stratification, targeted therapy, and prediction of response to therapy.

Immune checkpoint inhibitors (ICIs) have shown efficacy in treatment of metastatic TNBC, and immune profiling of tumors may predict efficacy of immunotherapies [7] The first immune checkpoint inhibitor FDA-approved in breast cancer atezolizumab, in combination with nab-paclitaxel in programmed death ligand 1 (PD-L1) positive TNBC, has shown encouraging efficacy [8]. Besides atezolizumab, other ICIs also showed efficacy in TNBCs [7, 9–11]. The role of immune check point inhibitors in metaplastic breast cancer is currently undergoing clinical investigation (NCT02834013).

The goal of this study is to understand the genomic and immune profiles of MpBC, and to study the association with clinical outcomes. An individual case of a metastatic MpBC patient carrying a PIK3CA mutation who had an exceptional response to everolimus is also reported here.

## Materials and methods

### Patient selection

A total of 21 cases of MpBC in patients who were diagnosed and treated from 1996 to 2014 were retrospectively identified. The eligibility criteria were pathological diagnosis of MpBC and availability of paraffin-embedded tumor tissue for analysis. The patient characteristics, disease characteristics, treatment history and survival data were collected. All procedures performed in studies involving human participants were in accordance with the ethical standards of the institutional and/or national research committee and with the 1964 Helsinki declaration and its later amendments or comparable ethical standards. All tumor specimens were identified through a City of Hope IRB-approved retrospective protocol from patients consented to City of Hope Biorepository Protocol IRB 07047 (COH does not provide a separate approval number). Written informed consent was obtained from all participants of this study.

### Pathology review

The archived tissue block from 20 surgical specimen and 1 metastatic biopsy were obtained. Hematoxylin and eosin (H&E) and immunohistochemical (IHC) stained slides were reviewed by two designated breast pathologists to confirm the diagnosis and morphological subtypes. The diagnosis of MpBC were based on the WHO 2012 Criteria [3]. In detail, MpBC were defined by the presence of squamous and/or mesenchymal (spindle or matrix-producing) elements, with or without coexisting ductal carcinoma in situ (DCIS) or conventional mammary-type invasive carcinoma. In cases lacking a conventional epithelial component, the diagnosis was confirmed using IHC staining, including low and high molecular weight cytokeratins and P63.

### Immune profiling

Tumor infiltrating lymphocyte (TIL) quantification was performed using the International TIL Working Group Criteria [12]. Immunohistochemistry for CD3, CD4, CD8, and immune cell PD-L1 was performed. For PD-L1 testing, Ventana PD-L1 clone SP263 was used. A total of > 1% of PD-L1 expression on immune cell and > 1+ by IHC was considered positive in this study [13, 14].

### Genomic analysis

NGS-based panel (Onco-48) analysis was performed using the Ion AmpliSeq^™^ Kit. Genomic DNA was extracted by micro-dissection of tissue from formalin-fixed, paraffin-embedded (FFPE) slides with minimum 30% tumor cellularity. A targeted DNA library was generated using the Ion AmpliSeq^™^ Cancer Hotspot Panel Kit and sequenced by semiconductor-based NGS technology on an Ion Torrent PGM. The Onco-48 Panel is designed to target 2800 mutations in 49 cancer genes: *ABL1*, *AKT1*, *ALK*, *APC*, *ATM*, *BRAF*, *CDH1*, *CDKN2A*, *CSF1R*, *CTNNB1*, *EGFR*, *ERBB2*, *ERBB4*, *EZH2*, *FBXW7*, *FGFR1*, *FGFR2*, *FGFR3*, *GNA11*, *GNAQ*, *GNAS*, *HNF1A*, *HRAS*, *IDH1*, *IDH2*, *JAK2*, *JAK3*, *KDR*, *KIT*, *KRAS*, *MET*, *MLH1*, *MPL*, *NOTCH1*, *NPM1*, *NRAS*, *PDGFRA*, *PIK3CA*, *PTEN*, *PTPN11*, *RB1*, *RET*, *SMAD4*, *SMARCB1*, *SMO*, *SRC*, *STK11*, *TP53*, *VHL*. NGS results were also obtained for two patients by clinical testing using FoundationOne®, a comprehensive genomic profile (CGP) that detects genomic alterations in more than 300 cancer-related genes and introns from more than 25 genes that are often rearranged or altered in solid tumors.

### Clinicopathologic analysis

Patient characteristics were captured including age, race, stage, chemotherapy and radiation therapy history, relapse free survival (RFS), and overall survival (OS). RFS was defined as date of surgery to date of first relapse, and OS was defined as date of surgery to date of death.

### Statistical analysis

Categorical variables were reported as counts (percent). Continuous data were reported as median (range). Descriptive survival analysis was performed using the Kaplan-Meier method, testing with the log-rank test. Estimates were reported along with their 95% confidence interval. Inference was drawn from Cox proportional-hazards regression modeling. Individual predictors were tested for significant differences using the Wald test. Statistical programming was done in the R programming language, version 3.4.1 [15]. Survival was estimated using the Survival add-on package, and plotted using the Survminer package [16, 17].

## Results

### Patients and clinical characteristics

Twenty-one eligible MpBC patients with survival data were included in this analysis. A total of 17 patients were tested with COH Onco-48 Gene Panel. Two patients’ results were obtained through clinical testing using FoundationOne® comprehensive genomic profile (CGP) for solid tumors. Two patients did not have adequate tissue for sequencing. The median age at time of diagnosis was 63 years (range 35–86) **(Table 1).** RFS at 5 years was 57% (95% CI 0.34–0.75), and OS at 5 years was 66% (95% CI 0.41–0.82). The subtypes of MpBC were: squamous (38.1%, 8/21), spindle (28.6%, 6/21), mixed squamous and spindle cell (14.3%, 3/21), spindle cell and mesenchymal (9.5%, 2/21), and mesenchymal (9.5%, 2/21). A total of 61.9% received both chemotherapy and radiation (13/21), 9.5% received chemotherapy alone (2/21), 9.5% received radiation alone (2/21), and 19.0% did not receive chemotherapy or radiation (4/21).

**Table 1:** Patient and treatment characteristics (n = 21).

Baseline Characteristics	n = 21	%
Age:	Median 63	Range 35–86
<50	5	24
50 to <70	7	33
≥ 70	9	43
Race:		
Non-Hispanic White	15	71
Hispanic	4	19
Asian	2	10
Breast Cancer Stage:		
I	4	19
II	16	76
III	1	5
Tumor grade:		
1	0	0
2	1	5
3	20	95
ER, PR, HER2 Status:		
ER-, PR-, HER2-	21	100
Metaplastic Breast Cancer Subtypes:		
Squamous	8	37
Spindle	6	29
Mixed squamous and spindle cell	3	14
Spindle cell and mesenchymal	2	10
Mesenchymal	2	10
Type of surgery:		
Lumpectomy	6	29
Mastectomy	15	71
Adjuvant Therapy:		
Anthracycline containing chemotherapy	11	53
Non-anthracycline chemotherapy	3	14
No (Patient Choice)	7	33
Adjuvant Radiation:		
Yes	15	71
No	6	29

### Mutations and survival analysis

**Fig 1:**
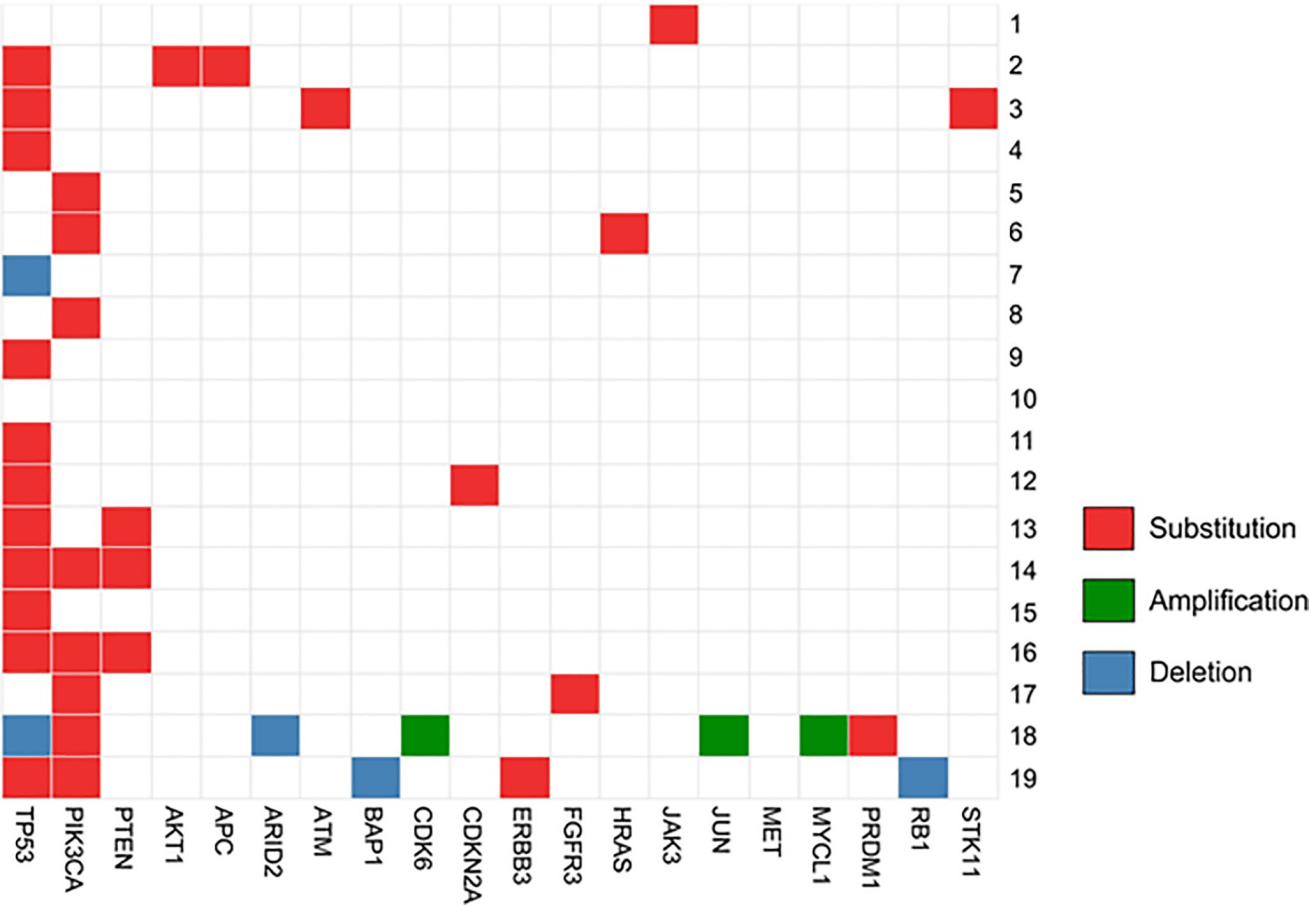
The most frequently altered genes in MpBC. Alterations include substitutions, amplifications, and deletions in MpBC patient cohort (n = 19).

**Fig 2:**
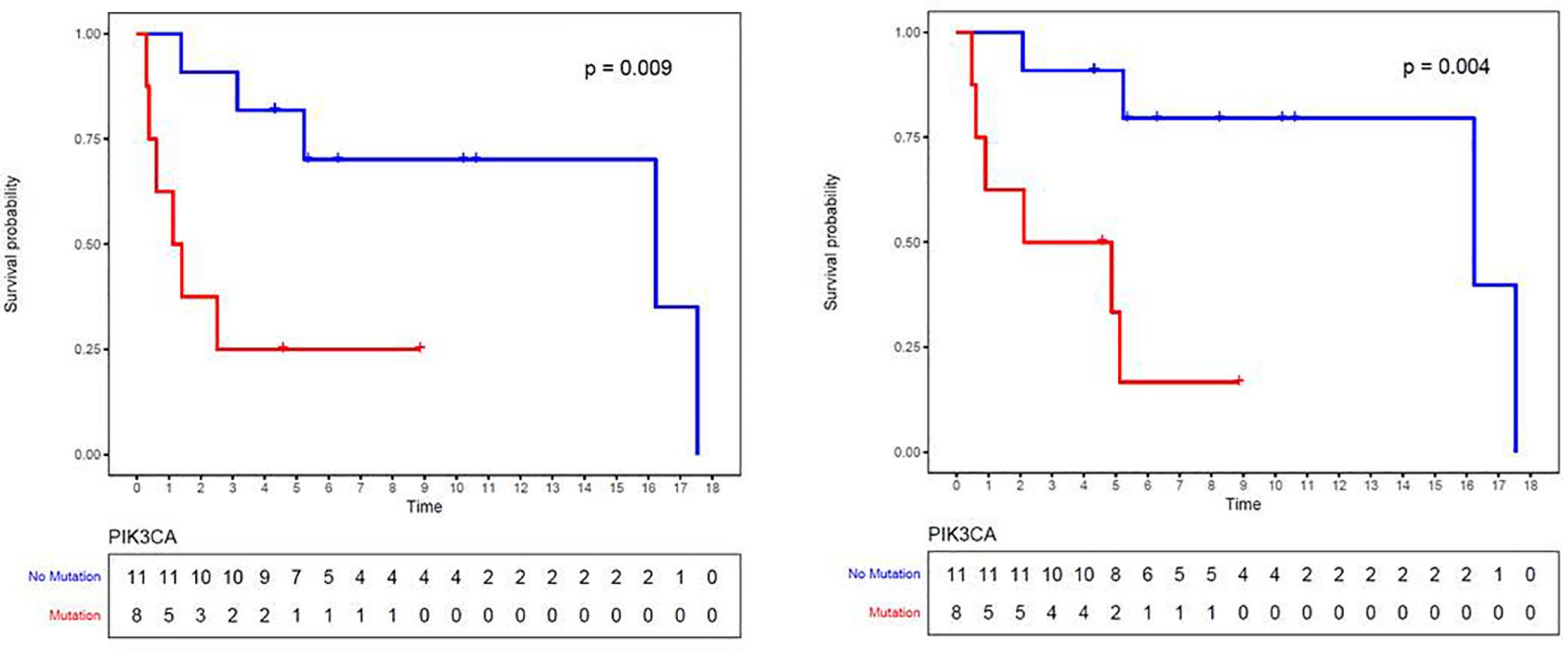
Kaplan Meier survival analysis for MpBC patients. A) RFS for patients with PIK3CA mutation (red) vs. no mutation (blue) (p = 0.009); B) OS for patients with PIK3CA mutation (red) vs. no mutation (blue) (p = 0.004).

Of the 49 cancer-driver genes studied, the most commonly altered genes in this cohort of MpBC included *TP53* (68.4%, 13/19), *PIK3CA* (42.1%, 8/19), and *PTEN* (15.8%, 3/19) **(Fig 1 and S1 Table).** In a univariate analysis, the association of *TP53*, *PIK3CA*, and *PTEN* mutations and breast cancer specific survival was determined. The median follow-up time of patients alive at date of analysis was 7.3 years (range 4.3–17.9). Recurrence free survival (RFS) at 5 years was 57% (95% CI 0.34–0.75) and overall survival (OS) at 5 years was 66% (95% CI 0.41–0.82). For patients with *PIK3CA* mutations, RFS and OS were significantly worse than for those without (HR 5.6, 95% CI 1.33–23.1 and HR 8.0, 95% CI 1.53–41.7, respectively) **(Fig 2)**. The other mutations were underpowered to be conclusive: *TP53* mutation associated with non-statistically significant reduction in hazard for RFS and OS (HR 0.66, 95% CI 0.16–2.63 and HR 0.56, 95% CI 0.13–2.34, respectively); *PTEN* was associated with non-statistically significant reduction in RFS and increase in hazard for OS (HR 0.79, 95% CI 0.10–6.44 and HR 1.54, 95% CI 0.17–13.8, respectively).

### Immune profiling of metaplastic breast cancer and survival analysis

**Fig 3:**
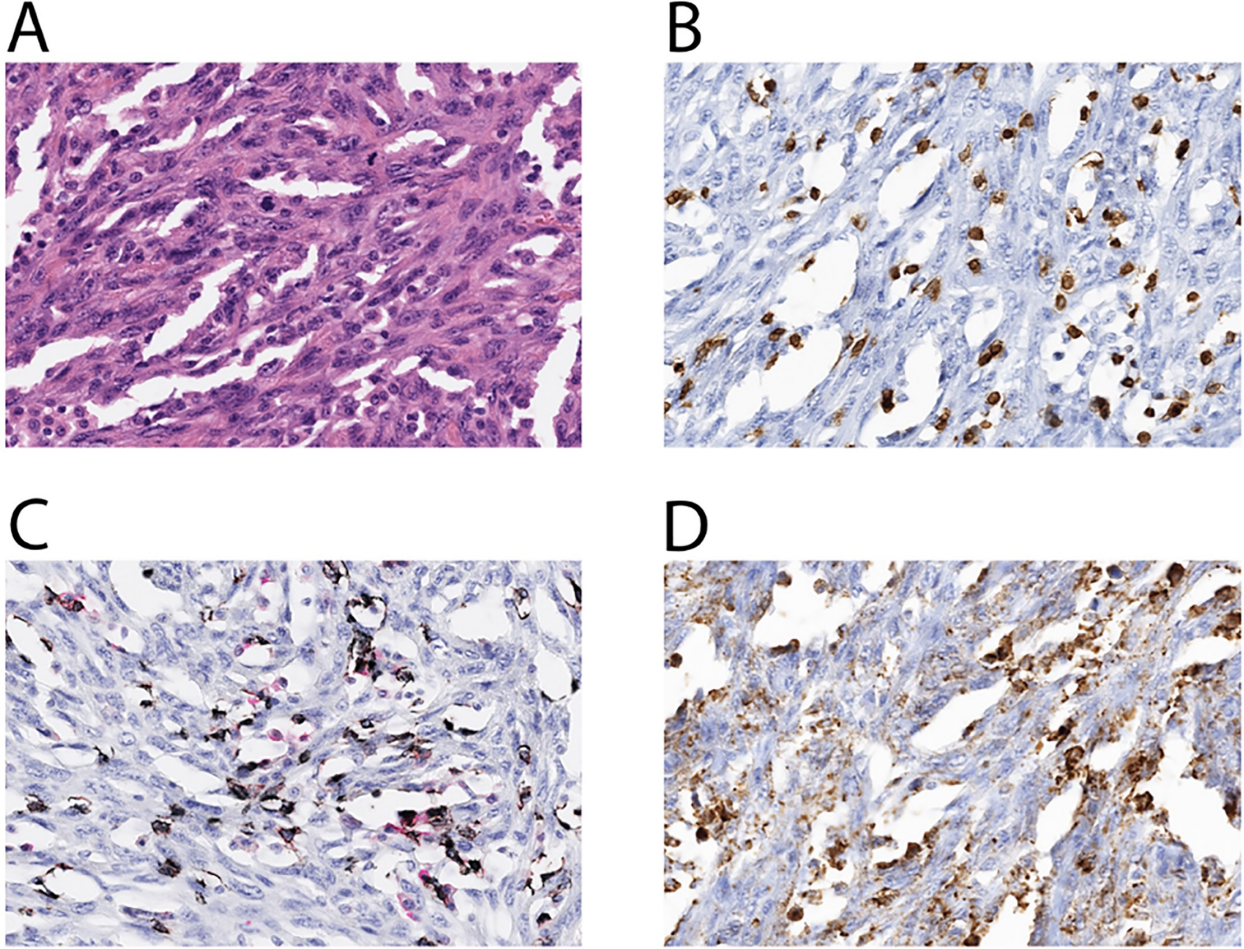
Representative IHC stain of CD3, CD4, CD8 and PD-L1. (A) H&E stained image of representative metaplastic carcinoma (spindle cell subtype) (original magnification 40X). (B) CD3 immunostain, highlighting T cells (original magnification 40X). (C) Combined CD8 (brown chromogen) and CD4 (red chromogen) immunostain (original magnification 40X). (D) PD-L1 immunostain (original magnification 40X).

Representative Immunohistochemistry (IHC) for CD3, CD4, CD8, and PD-L1 is shown in **Fig 3 and S1 Table**. Analysis was performed on 14 samples, due to lack of tumor tissue for 7 samples. A total of 50% had CD3 positive cells ≥ 10% (7/14), and 35.7% had CD8 positive T cells ≥ 10% (5/14). Only 1 tumor had ≥ 10% CD4-positive T cells. Positive PD-L1 (Ventana PD-L1 clone SP263), defined by greater than 1% of cells stained 1+ by IHC, was observed in 50% samples (7/14). The PD-L1 expression was a significant predictor of poor RFS and OS when tested as a continuous variable. Specifically, an absolute increase in PD-L1 expression of 1% was associated with worse RFS and OS (HR 1.08, 95% CI 1.01–1.16 and HR 1.05, 95% CI 1.0–1.11, respectively, p< 0.05 for both) (**S1 Fig**). When dichotomizing the data based on best cut-point for display purposes, we considered patients with tumor PD-L1 > 10% (solid line) vs. PD-L1 ≤ 10% (dashed line). In this case, the empirical p-value (unadjusted for the multiple cut-point testing) is reported, although the proper statistical test is based on treating PD-L1 expression as a continuous marker (p<0.05).

### A case of PI3K-targeted therapy for metaplastic breast cancer

**Fig 4:**
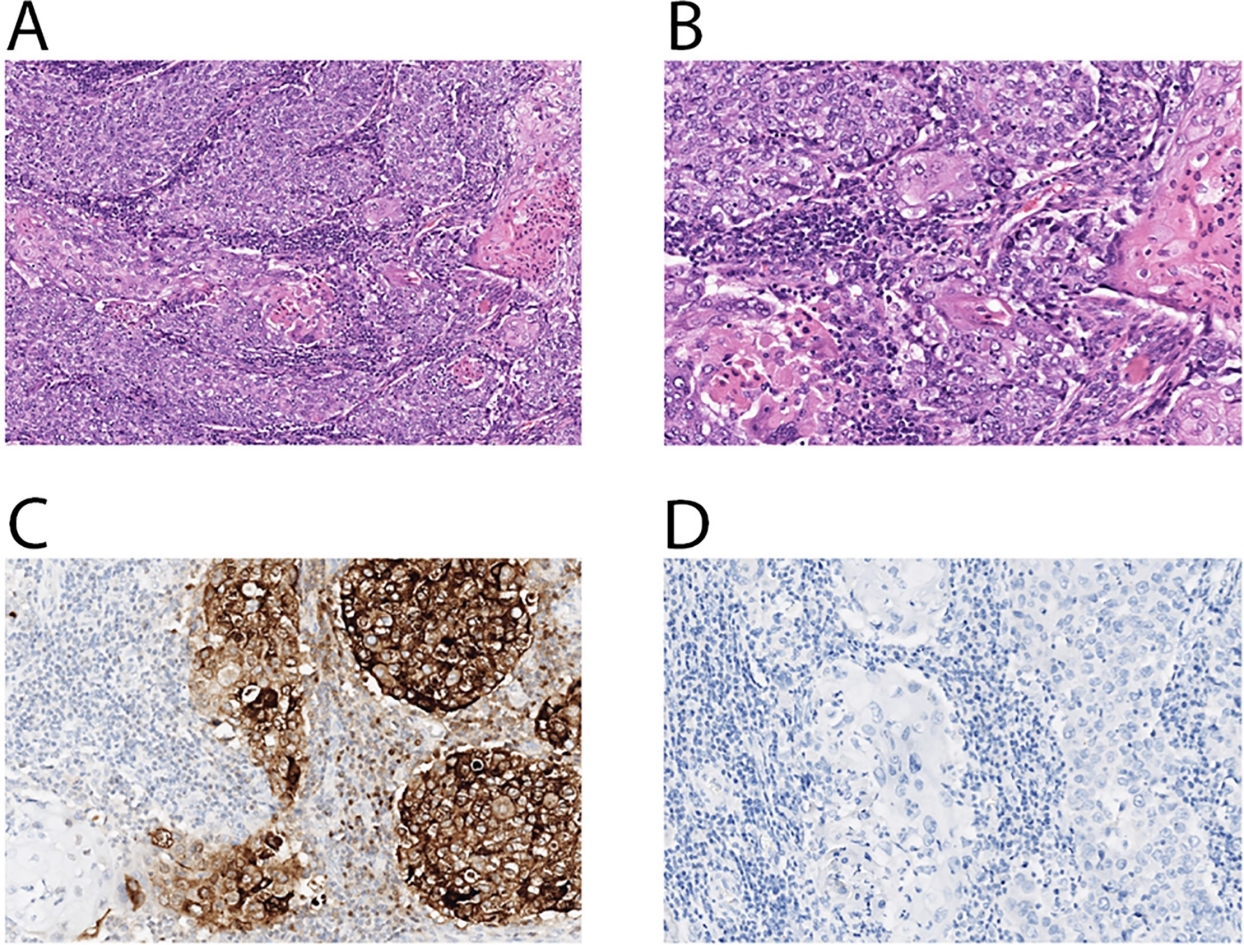
Photomicrographs of lung biopsy demonstrating metastatic MpBC with squamous differentiation invading lung parenchyma. (A) Hematoxylin-eosin staining (original magnification 4X), (B) Hematoxylin-eosin staining (original magnification 20X), (C) IHC showing tumor cells are positive for GCDFP-15 (original magnification 20X), and (D) IHC showing tumor cells are negative for TTF-1 (original magnification 20X), consistent with MpBC. MpBC, metaplastic breast cancer; IHC, immunohistochemistry; GCDFP-15, gross cystic disease fluid protein 15; TTF-1, thyroid transcription factor-1.

**Fig 5:**
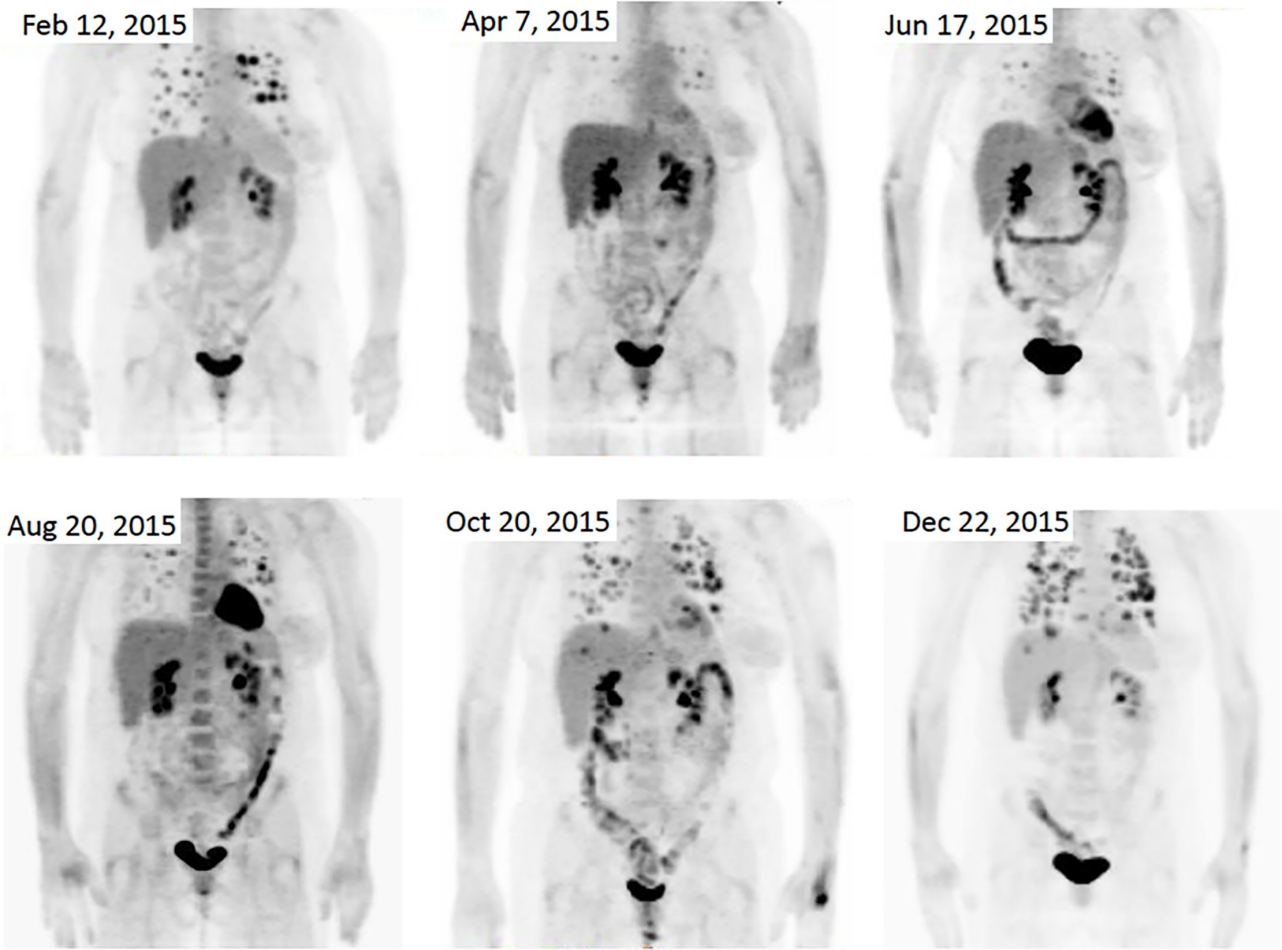
Treatment response by PET-CT. (A) Baseline imaging demonstrating pulmonary metastases (February 2015); (B) Imaging after 2 months of treatment with everolimus, demonstrating tumor regression (April 2015); (C, D) Persistent responses; (E, F) Disease progression.

We identified an exceptional responder treated with mTORC1 inhibitor everolimus. A 49-year-old woman underwent a right modified radical mastectomy for a stage II high grade MpBC in March 2011. She received standard adjuvant chemotherapy with doxorubicin, cyclophosphamide and paclitaxel followed by radiation. In September 2013, a solitary lung nodule was identified on routine chest x-ray and was resected via thoracotomy, which confirmed a 2.5 cm metastatic MpBC (**Fig 4**). FoundationOne® genomic test identified *TP53* p190del, *PIK3CA* C420R, *CDK6* amplification, *MYCL1* amplification, and *JUN* amplification. Patient received capecitabine with poor response. Restaging PET-CT in February 2015 demonstrated multiple bilateral pulmonary nodules with high standardized uptake value (SUV) of 7.6–16.7 (**Fig 5A**). Everolimus (10mg daily) was initiated in February 2015 with significantly reduced cough and shortness of breath, and improved performance status within a week. A restaging PET-CT in April 2015 confirmed marked improvement of pulmonary metastases (**Fig 5B**). The patient was continued on treatment until December 2015 and achieved 8 months of disease control by PET-CT imaging (**Fig 5C–5F**).

## Discussion

Chemotherapy remains the treatment of choice for MpBC and the prognosis is extremely poor with a median overall survival of 8 months in patients with metastatic MpBC [18, 19]. There is an urgent need to identify novel molecular targets and develop clinical trials for treatment of this rare disease. In this study, we identified an association between PIK3CA mutation and poor RFS and OS in a cohort of patients with MpBC. We reported a patient with metastatic MpBC with an exceptional response to everolimus, a PI3K-mTOR targeted therapy. The current findings are hypothesis-generating and may indicate a role of PI3K-targeted therapy in treating MpBC.

NGS can provide unique insight into the underlying biology of MpBC and may inform clinical decision making. In our study, *PIK3CA* mutation was found in 42.1% of MpBC patients. Other studies have reported a similar finding of 23–48% of PIK3CA mutations in MpBCs [20–22]. In a recent study by Ng et al., *PIK3CA* (29%), *PIK3R1* (11%), *ARID1A* (11%), *FAT1* (11%), and *PTEN* (11%) were identified a cohort of 35 MpBCs (23). Compared with invasive ductal TNBCs, MpBCs significantly more frequently harbored mutations in *PI3K/AKT/mTOR* pathway–related (57% vs. 22%) and canonical Wnt pathway–related (51% vs. 28%) genes [23]. Collectively, these studies provide evidence of heterogeneity of MpBC and indicate that PIK3CA/AKT/mTOR pathway could serve as an important target for therapy. Although *TP53* is one of the most commonly identified mutations in human breast cancer, targeting p53 has been challenging [24]. *PIK3CA* mutations are common in breast cancer with a reported rate of 25–40% of all breast cancers. The *PIK3CA* mutation C420R is an activating mutation predicted to be oncogenic. *PIK3CA* encodes the p110-alpha catalytic subunit of phosphatidylinositol 3-kinase (PI3K), and this pathway is involved in cell growth, proliferation, differentiation, motility and survival. Activating mutations may predict sensitivity to inhibitors of PI3K, AKT, or mTOR, including mTOR inhibitors everolimus and temsirolimus. In a subset analysis of a phase I study, metastatic MpBC treated with mTOR inhibitor temsirolimus plus liposomal doxorubicin and bevacizumab had an encouraging response rate of 42% [25, 26]. Moulder *et al*. recently reported a response rate of 25% in 23 patients with metastatic MpBC treated with six different temsirolimus-based chemotherapy regimens [19]. A prospective clinical trial is needed to verify this finding.

Immunotherapies such as checkpoint inhibitors are undergoing rigorous investigation in clinical trials for treatment of TNBC [7, 10, 11, 27]. Joneja *et al*. recently reported enriched PD-L1 expression in 46% of MpBCs [22]. Adams *et al*. reported a case of dramatic response of MpBC to the combination of nab-paclitaxel and immune check point inhibitor pembrolizumab [28, 29]. In our study, 46.7% of MpBCs have >1% of PD-L1 expression and tumor PD-L1 expression is associated with poor 5-year RFS and OS. PD-L1 has been shown to be a predictive biomarker for response to immune checkpoint inhibitors [30]. Our study provides additional evidence that immune checkpoint inhibitors may play an important role in the treatment of MpBC.

Due to the rarity of MpBC, the current study is limited by its small sample size, retrospective nature, single center experience and limited 49-gene panel. A larger sized study with inclusion of whole exome sequencing and RNA sequencing will be required to verify the current finding. We are currently working with the Oncology Research Information Exchange Network (ORIEN) to build a genomic database of MpBC.

## Conclusion

In this study, we report a unique cohort of MpBC with genomic and immune characterization. *PIK3CA* mutation and PD-L1 expression were associated with worse survival in this cohort of patients with MpBC. The case of exceptional response to everolimus reported here demonstrates how next-generation sequencing can identify effective therapies in patients with uncommon breast cancer histologies, for whom evidenced-based treatments are currently lacking.

## Supporting information

S1 Fig Kaplan-Meier graphs for number of patients at risk by average % tumor PD-L1. (A) Relapse-free survival (RFS); and (B) Overall survival (OS). Graphs are dichotomized ≤ 10% PD-L1 (dotted line) vs. > 10% PD-L1 (solid line).
(TIF)

S1 Table Patient polymorphisms, genomic alterations, and tumor infiltrating lymphocytes (N = 19). (DOCX)
