# Supplementary figures and images for: Mutation and immune profiling of metaplastic breast cancer: Correlation with survival
Source: PLoS One. 2019 Nov 6;14(11):e0224726. doi: 10.1371/journal.pone.0224726 (PMC6834262; doi:10.1371/journal.pone.0224726)

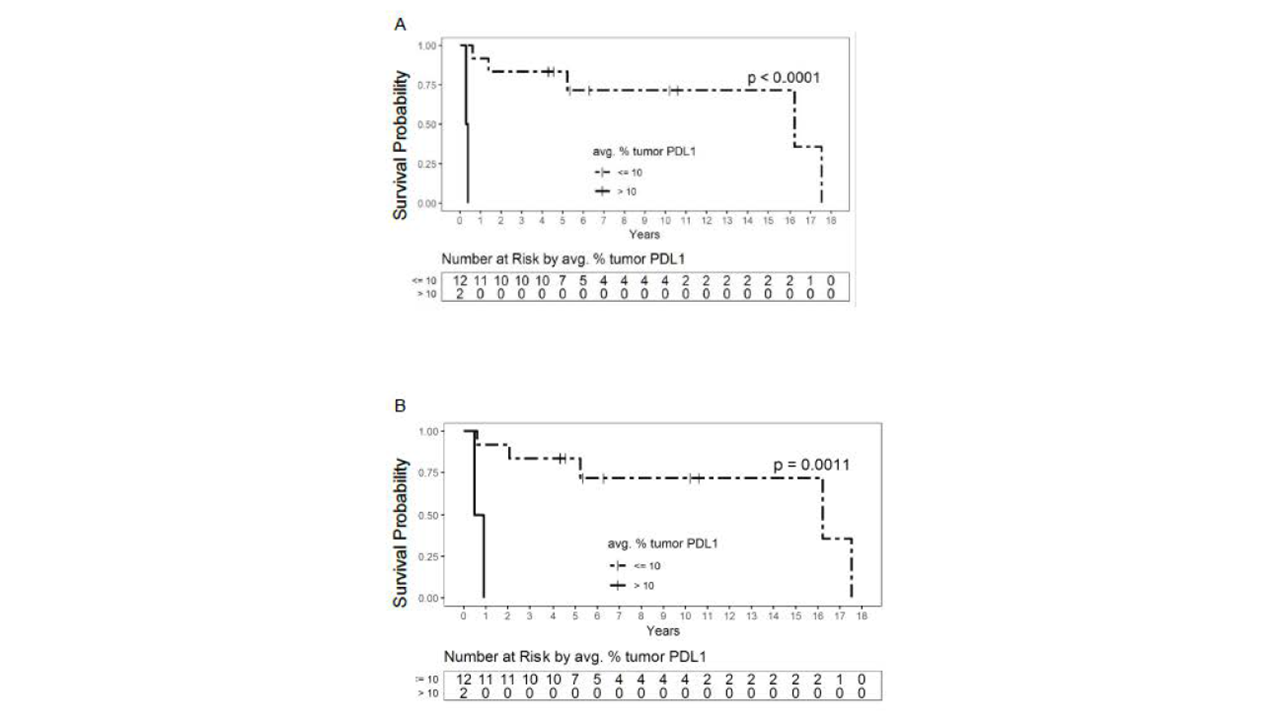

Supplement: S1 Fig — (A) Relapse-free survival (RFS); and (B) Overall survival (OS). Graphs are dichotomized ≤ 10% PD-L1 (dotted line) vs. > 10% PD-L1 (solid line). (TIF) [file pone.0224726.s001.tif]
