# Supplementary material for: Mutation and immune profiling of metaplastic breast cancer: Correlation with survival
Source: PLoS One. 2019 Nov 6;14(11):e0224726. doi: 10.1371/journal.pone.0224726 (PMC6834262; doi:10.1371/journal.pone.0224726)
Supplement: S1 Table — (DOCX) [file pone.0224726.s002.docx]

**Supplemental Table 1:** Patient polymorphisms, genomic alterations, and tumor infiltrating lymphocytes (N=19)

| Pt | Polymorphism | | | | | Clinically significant genomic alterations | | | | Average % tumor infiltrating lymphocytes | | | |
| --- | --- | --- | --- | --- | --- | --- | --- | --- | --- | --- | --- | --- | --- |
|  | TP53 | PIK3CA | KDR | KIT | MET | TP53 | PIK3CA | PTEN | Other | Ave % CD3 | Ave % CD4 | Ave % CD8 | Ave % PD-L1 |
| 1 |  |  |  |  |  |  |  |  | JAK3, V7221 | 8 | 0 | 5 | 5 |
| 2 | P72R |  | Q472H | M541L |  | G266R |  |  | APC, E1317Q; AKT1, E17K | 5 | 3 | 2 | 2 |
| 3 | P72R | I391M |  |  |  | G245D |  |  | ATM, R337C; STK11, F354L | N/A | N/A | N/A | N/A |
| 4 |  |  |  |  |  | R273H |  |  |  | 5 | 0 | 2 | 1 |
| 5 | P72R |  | Q472H |  |  |  | H1047R |  |  | N/A | N/A | N/A | N/A |
| 6 | P72R |  | Q472H |  |  |  | E545K |  | HRAS, Q61H | 15 | 0 | 15 | 35 |
| 7 | P72R |  | Q472H |  |  | Leu252del |  |  |  | 1 | 1 | 1 | 1 |
| 8 | P72R |  |  |  |  |  | H1047R |  |  | N/A | N/A | N/A | N/A |
| 9 | P72R |  | Q472H |  |  | Tyr126Cys |  |  |  | 3 | 2 | 1 | 3 |
| 10 | P72R | I391M |  | M541L |  |  |  |  |  | 10 | 0 | 5 | 1 |
| 11 | P72R | I391M |  |  |  | C229Yfs*10 |  |  |  | 8 | 2 | 8 | 1 |
| 12 |  |  |  |  | R988C | P152AFs*14 |  |  | CDKN2A, H123Q | 30 | 20 | 10 | 10 |
| 13 | P72R |  | Q472H |  |  | R248W |  | PTEN, Y68H |  | 25 | 5 | 20 | 5 |
| 14 | P72R |  |  |  |  | R248W | H1047R | PTEN, T319Nfs*6 |  | 10 | 0 | 10 | 1 |
| 15 | P72R |  |  |  |  | R306* |  |  |  | 10 | 0 | 5 | 1 |
| 16 | P72R |  |  |  |  | H214Qfs*7 | Q546R | PTEN, Y240* |  | 5 | 1 | 4 | 1 |
| 17 |  |  |  |  |  |  | E545K |  | FGFR3, F384L | 15 | 0 | 15 | 50 |
| 18 | N/A | N/A | N/A | N/A | N/A | P190del | C420R |  | CDK6, amp; MYCL1, amp; splice site 1121-2_1133del15; JUN, amp; PRDM1, A312fs*66 | N/A | N/A | N/A | N/A |
| 19 | N/A | N/A | N/A | N/A | N/A | R273H | E545K |  | ERBB3 V104M; BAP1 loss; RB1 loss exons 1-17 | N/A | N/A | N/A | N/A |
